# Trophic effects of Bti-based mosquito control on two top predators in floodplain pond mesocosms

**DOI:** 10.1007/s11356-024-34124-w

**Published:** 2024-07-05

**Authors:** Verena Gerstle, Eric Bollinger, Alessandro Manfrin, Sebastian Pietz, Sara Kolbenschlag, Alexander Feckler, Martin H. Entling, Carsten A. Brühl

**Affiliations:** 1 Institute for Environmental Sciences, iES Landau, RPTU Kaiserslautern-Landau, Fortstraße 7, D-76829 Landau, Germany; 2 Eußerthal Ecosystem Research Station, RPTU Kaiserslautern-Landau, Birkenthalstraße 13, D-76857 Eußerthal, Germany

**Keywords:** *Bacillus thuringiensis* var. *israelensis*, Newt, Dragonfly, Chironomid, Stable isotope analysis, Neutral lipid fatty acids

## Abstract

Chironomid (Diptera: Chironomidae) larvae play a key role in aquatic food webs as prey for predators like amphibian and dragonfly larvae. This trophic link may be disrupted by anthropogenic stressors such as *Bacillus thuringiensis* var*. israelensis* (Bti), a biocide widely used in mosquito control. In a companion study, we recorded a 41% reduction of non-target larval chironomids abundance in outdoor floodplain pond mesocosms (FPMs) treated with Bti. Therefore, we examined the diet of two top predators in the FPMs, larvae of the palmate newt (Salamandridae: *Lissotriton helveticus*) and dragonfly (Aeshnidae: predominantly *Anax imperator*), using bulk stable isotope analyses of carbon and nitrogen. Additionally, we determined neutral lipid fatty acids in newt larvae to assess diet-related effects on their physiological condition. We did not find any effects of Bti on the diet proportions of newt larvae and no significant effects on the fatty acid content. We observed a trend in Aeshnidae larvae from Bti-FPMs consuming a higher proportion of large prey (Aeshnidae, newt, damselfly larvae; ~42%), and similar parts of smaller prey (chironomid, mayfly, Libellulidae, and zooplankton), compared to controls. Our findings may suggest bottom-up effects of Bti on aquatic predators but should be further evaluated, for instance, by using compound-specific stable isotope analyses of fatty acids or metabarcoding approaches.

**Supplementary Information:**

The online version contains supplementary material available at 10.1007/s11356-024-34124-w.

## Introduction

Chironomids are a dipteran family of non-biting midges, whose larvae play a crucial role in the food webs of aquatic ecosystems. Their high abundance in wetlands makes them an important food source for aquatic and terrestrial predators (Armitage et al. 1995). Despite them being non-target organisms, the use of the mosquito control biocide *Bacillus thuringiensis* var*. israelensis* (Bti) in wetlands has been shown to reduce the abundance of aquatic chironomid larvae (Allgeier et al. 2019a; Gerstle et al. 2023). Bti is a naturally occurring soil bacterium that produces crystal proteins toxic to larvae of the dipteran suborder Nematocera, which includes families of biting Culicidae, Simuliidae, and Ceratopogonidae, as well as non-biting Chironomidae. Once ingested by the organism, the crystal proteins bind to receptors in the midgut, causing cell lysis and ultimately the death of the larvae (Becker 1997; Ben-Dov 2014).

Due to its specific mode of action, direct lethal effects of Bti on non-target organisms, apart from chironomids, are unlikely (as reviewed by Boisvert & Boisvert 2000, and Brühl et al. 2020), which leads to a labelling of Bti as an environmentally friendly biocide. However, the impact of Bti on non-target chironomids can potentially shift food webs, inducing bottom-up effects on aquatic predators (Allgeier et al. 2019b; Gerstle et al. 2023). In an outdoor mesocosm experiment involving macroinvertebrate communities and two predators, namely dragonfly (Odonata: Aeshnidae) and newt (Urodela: Salamandridae) larvae, stable isotope analyses of carbon (C) and nitrogen (N) revealed that chironomids were the preferred food source of newts, constituting over 56% of their diet (Allgeier et al. 2019b). Additionally, mortality of newts was 27% higher in mesocosms treated with Bti, suggesting increased intraguild predation (competition and predation of species with similar trophic niches; Holt & Polis 1997). Similarly, a recent study by Gerstle et al. (2023) reported a 41% abundance reduction of chironomid larvae in Bti-treated floodplain pond mesocosms (FPMs), as well as a 54% reduced emergence of the Odonata family Libellulidae. These findings further strengthened the hypothesis of intraguild predation in dragonflies due to decreased chironomid availability. In general, predator coexistence strongly depends on prey density (Preston et al. 2018; Takatsu 2022). Therefore, changes in their diet are of considerable interest when assessing food webs.

A different effect pathway of Bti for the aquatic food web is by altering soil microbial communities (as reviewed in Belousova et al. 2021). These alterations can affect detritus-processing organisms, such as protozoans and nematodes (McKie et al. 2023). McKie et al. (2023) used stable isotope analysis of C and N and suggested an increased food chain length in Bti-treated floodplains due to potential feeding by protozoans on decaying mosquito larvae. These findings further highlight the complexity of food webs and consequently the need for field experiments with natural communities but yet in a replicated comparable test design.

To address this, we investigated the diets of two top predators, larval palmate newts (*Lissotriton helveticus*) and Aeshnidae (mostly *Anax imperator*), in 12 outdoor FPMs, half of them exposed three times to the maximum field rate of Bti between April and May 2020. We used stable isotope analyses of C and N to understand how the diet of the two top predators responds to a reduction of larval chironomid availability as prey. Due to the 41% reduction of chironomid larvae in Bti-treated FPMs recorded by Gerstle et al. (2023) in the same experiment, we hypothesized that larval newts and Aeshnidae switch to other prey than chironomids, such as mayflies, zooplankton or other predators, and smaller conspecifics (intraguild predation). Therefore, we hypothesized a higher trophic position of newts and Aeshnidae in Bti-FPMs. We also hypothesized a larger niche size in Bti-FPMs because of a more generalist feeding activity and consumption of alternative prey. To obtain information on the physiological consequences of an altered diet, we included the analyses of neutral lipid fatty acids (NLFAs) in newt larvae as vertebrate top predators. NLFAs are mostly utilized as storage lipids in tissues (Turkish and Sturley 2009), which gives further insight into the utilization of the ingested prey and the newts’ diet, i.e., retention of fatty acids (FAs) (Kainz et al. 2004; Twining et al. 2016, 2021) and, in turn, also the nutritional value for aquatic and terrestrial higher-level carnivores, e.g. birds, large ground beetles, predatory fish.

## Material and methods

### Study site

The experiment was conducted in twelve FPMs at the Eußerthal Ecosystem Research Station (49°15′14″N, 7°57′42″E; RPTU Kaiserslautern-Landau) in the Palatinate Forest in Southwest Germany. The FPMs were constructed in 2017 and were allowed for natural colonization by organisms from nearby freshwater habitats (for details see Stehle et al. 2022). Each FPM (23.5 m × 7.5 m) is equipped with an inlet connected to the adjacent small stream Sulzbach and an adjustable outlet, with which the water depth can be regulated. In its initial state, the water level is 30 cm at the deepest point and gradually fades into a shallow floodplain area as well as a terrestrial shore at one side. The vegetation in the aquatic part predominantly consists of waterweeds, coontails, and filamentous green algae, while the shores are mostly covered by emergent plants such as bulrushes and soft rush.

The FPMs are a breeding habitat for various amphibians, predominantly European common frogs (*Rana temporaria*), European common toads (*Bufo bufo*), and palmate newts (*Lissotriton helveticus*). Since temporarily high densities of amphibian larvae strongly influence aquatic food web structures, we aimed for an even distribution of amphibian larvae across the twelve FPMs. Therefore, we installed an amphibian fence around the FPMs to control the immigration of adult frogs and toads and placed bottle traps inside the FPMs to capture adult newts (Griffiths 1985; for more information see Gerstle et al. 2023). To ensure that amphibian larvae are not completely excluded from the food web, we introduced two to three toad egg strings, five frog egg clutches of similar weight, and 20 adult palmate newts (ten male and ten female) to each FPM 1 week prior to the start of the experiment.

### Bti application and flooding

In floodplains of the Upper Rhine Valley in southwest Germany, inundated wetlands are treated multiple times per year on a large scale to control nuisance by the mass emergence of the floodwater mosquito (*Aedes vexans*; Becker 2003). Since inundation triggers the hatching of the larvae, Bti applications in these wetlands are linked to temporary floodings. To mimic a realistic Bti application scenario, we flooded the FPMs three times by increasing the water level from 30 to 50 cm from mid-April to the end of May 2020. On the third day of flooding (for dates see Table S1), we applied the maximum field rate of VectoBac WDG (2.88 × 10^9^ ITU/ha; Valent BioSciences, IL, USA) to every other FPM using a knapsack sprayer (Prima 5, Gloria, Germany). The maximum field rate of Bti is applied when the water is deeper than 10 cm and/or late instar mosquito larvae are targeted (Becker 1997).

As there is currently no analytical method available to quantify sterilized Bti in the environment, we implemented a biotest using mosquito larvae (*Culex* sp.) to validate the efficacy of the Bti treatment (i.e., at least 90% mortality of mosquito larvae within a week; for more information on the biotest, see Gerstle et al. 2023).

### Sampling

For stable isotope analyses, we sampled newt larvae (*L. helveticus*) and Aeshnidae larvae (predominantly *A. imperator*) as well as their potential resources 3 weeks after the last Bti application (13-16 June 2020, Table S1). Resources included larval Libellulidae, chironomids, mayflies, and zooplankton. Depending on the number of organisms present in the FPM (for sample size, see Table S2), we aimed to sample ten individuals of similar sizes for each consumer and five subsamples of each resource per FPM (total of 10 FPMs used for statistical analyses due to limited availability of consumers; Table S2). Sampling of similar sizes of consumers was done to reduce possible effects of size-selective prey selection for diet analyses and did not represent the mean body size of consumers in the FPMs. To collect newt larvae and macroinvertebrates, we used a 500-µm-mesh dip net. Zooplankton was sampled by dragging a 55-µm-mesh dip net through the water column of the entire FPM. We applied a non-quantitative sampling until all relevant resources were collected. All organisms were kept in filtered pond water overnight (mesh size 55 µm) to allow the release of gut contents. Thereafter, samples were rinsed with distilled water and shock-frozen in liquid nitrogen. All samples were stored at −20 °C until further processing.

For FA analyses, we collected, if possible, five *L. helveticus* larvae from four FPMs using a dip-net (two control and two Bti-treated FPMs). We sampled larvae of similar sizes and developmental stages from four instead of 12 FPMs, since we were not able to sample enough larvae of comparable body size in each FPM to allow for statistical investigation. Sampling was not done This procedure was further motivated by, firstly, newts’ diet dependency on their body size (size-selective predation) and, secondly, energy reserves changing during development and metamorphosis (Crump 1981; Pfab et al. 2020). The sampling of larvae for FA analyses was conducted simultaneously with the sampling of larvae for stable isotope analyses, and samples were treated as described above but stored at −80 °C.

### Stable isotope analysis

We freeze-dried all samples until complete dryness (at least 48 h). For consumers, we dissected the body part with the highest content of muscle tissue, namely the tail for newt larvae and the thorax for Aeshnidae (Allgeier et al. 2019b; Seifert and Scheu 2012). Dried samples were homogenized in 1.5-mL Eppendorf tubes using metal beads and a tissue lyzer (Retsch, MM 301, Germany). For each sample, a dry mass of 0.6 ± 0.1 mg homogenized sample was packed into a tin capsule (5 × 9 mm, IVA, Meerbusch, Germany) using an ultrafine balance (precision: 0.1 µg; Sartorius, Germany). We pooled several individuals into one sample if an individual organism did not yield enough biomass (i.e., chironomids, mayflies and zooplankton). Stable isotope ratios of C and N were determined using an elemental analyzer (EA, Flash 2000 HT, Thermo Scientific, Bremen, Germany) coupled to an isotope ratio mass spectrometer (IRMS, Delta V Advantage, Thermo Scientific, Bremen, Germany). The values are reported in delta notation:

1 $$\delta X=\left(\frac{{R}_{sample}}{{R}_{standard}}-1\right)*1000\permille$$

with *δX* being *δ*^*13*^*C* or *δ*^*15*^*N*, and *R*_*sample*_ and *R*_*standard*_ the abundance ratios of the heavy to the light isotope of the sample and the international standard (i.e., Vienna Pee Dee Belemnite for C and atmospheric air for N, respectively). As a working standard, casein was measured in duplicate every ten samples with a precision of <0.06‰.

### NLFA analyses

Although the focus of the study is stable isotope analyses of C and N, we decided to include quality parameters for the vertebrate predator. Therefore, we examined whole-body neutral lipid fatty acid (NLFA) contents of newt larvae from control-FPMs (*n*=2; 7 larvae in total) and Bti-FPMs (*n*=2; 10 larvae in total). Samples were freeze-dried for 48 h and weighed (13.49 ± 4.80 mg (mean ± SD); Fig. S2). As described in Konschak et al. (2020) and Pietz et al. (2023), we extracted NLFAs from whole-body samples (manually crushed using glass pasteur pipettes) using a chloroform to methanol to Milli-Q water mixture (1:2:0.8; v:v:v). In a second step, chloroform and Milli-Q water were added to obtain a final mixture of 2:2:1.8 (v:v:v). As an internal standard, we added a triacylglycerol with three deuterated 18:0 FAs (Tristearin-D105; Larodan). Lipids were extracted from the samples by overnight storage at 4 °C. The following day, neutral lipids were separated from phospholipids and glycolipids via solid-phase extraction by eluting in 4 mL of chloroform and pre-conditioned (4 mL of chloroform) polar-modified polystyrene/divinylbenzene copolymer cartridges (Chromabond® easy polypropylene columns; Machery-Nagel). Samples were dried at 40 °C under a stream of nitrogen gas and afterwards re-dissolved in 100 µL of dichloromethane. To hydrolyze lipids and methylate fatty acids to fatty acid methyl esters (FAMEs), we added trimethylsulfonium hydroxide (Sigma-Aldrich).

FAMEs were analyzed using gas chromatography with flame-ionization detection (GC-FID; Trace GC Ultra; Thermo Fisher Scientific) and a Restek FAMEWAX capillary GC column (30 m × 0.25 mm × 0.25 µm film thickness), and helium (1.4 mL/min) as carrier gas. Based on different retention times, FAMEs were determined using FAME standards (37-component FAME Mix; Supelco CRM47885) and quantified as µg FA per mL using an external standard calibration. The FAME of the internal standard (Methyl D-35 Octadecanoate; Larodan) was added directly to the standards of the calibration series in the same concentration as the lipid internal standard (Tristearin-D105) in the samples. Using the recovery rate of the internal standard and blank correction, concentrations of FAs were adjusted for inaccuracies of the instrument. To obtain FA concentration in µg FA per mg dry weight, we normalized the corrected FA concentration by the total volume and dry weight of the samples.

### Statistical analyses

#### Stable isotope analyses

To test for a possible influence of the body size on the isotopic composition of the consumers, differences in body mass of Aeshnidae and newts were investigated using generalized linear mixed models (GLMM) from the package “lme4” (version 1.1.27.1, Bates et al. 2014) for R (version 4.1.1, R Core Team 2021). Prior to GLMM, we checked for normality and homoscedasticity using visual inspection of quantile-quantile plots and Levene’s test (“car” package, version 3.0-11; Fox and Weisberg 2019), respectively. We then applied Bti treatment as a fixed effect to the GLMM and FPM identity as a random effect to account for pseudo-replication within the FPMs. Post-hoc comparisons were conducted using the *emmeans* (estimated marginal means) function from the package “emmeans” (version 1.7.0; Lenth (2016) for R.

Before analyses of diet proportions, we calculated the trophic levels (TL) for Aeshnidae and newts (Eq. 2):

2 $${TL}_{consumer}={TL}_{base}+ \frac{{{d}^{15}N}_{consumer} - mean ({{d}^{15}N}_{base})}{{TEF}_{d15N}}$$

with a primary consumer, i.e., zooplankton as baseline organism (TL = 2) and consumer-specific trophic enrichment factors (TEF). For newt-specific TEFs, we used 0.1 ± 0.4‰ for δ^13^C and 2.3 ± 0.4‰ for δ^15^N (Cloyed et al. 2015) and for Aeshnidae, we used 0.8 ± 0.2‰ and 0.9 ± 0.2‰ (Brauns et al. 2018). Since there are no third-level carnivorous consumers (TL 5) in the FPM system, we removed samples with TL higher than 4, as this may be the result of a methodological artifact (six newts removed) and would most likely confound diet analyses.

We grouped sources by k-means clustering with the optimal number of clusters being determined based on within sum of squares and the structure of the polygon. The reduction of a number of sources (i.e., clusters) in the model (Phillips et al. 2014) lowers its underdetermination. The diet proportions of newt and Aeshnidae larvae were determined using Bayesian mixing models for each FPM (chain length = 50,000; burn-in = 5000; thinning = 100; chains = 4; resid_err = TRUE; process_err = TRUE) using the package “MixSIAR” (version 3.1.12; Stock et al. 2018) for R. We applied the abovementioned consumer-specific TEFs to the resources to account for trophic enrichment that is systematical increase in stable isotopes throughout the food web. To estimate the relative niche sizes (i.e., the resources an organism can utilize) of newt and Aeshnidae larvae, we calculated Bayesian standard ellipse areas (SEA) using the package “SIBER” (version 2.1.6; Jackson et al. 2011) for R and normalized to the SEA of resources to allow comparison between FPMs. We determined the trophic position of newts and Aeshnidae in the food web using the package “tRophicPosition” (version 0.8.0; Quezada-Romegialli et al. 2018) for R and the same TEFs applied before (see Eq. 2).

#### Fatty acid analyses

We grouped single FAs into FA groups of saturated FAs (SFA), monounsaturated FA (MUFA), and polyunsaturated FA (PUFA), and physiologically relevant omega-3 and omega-6 PUFAs (for grouping, see Table 1). We removed one outlier (one newt larvae from the control; above the 1.5 interquartile range) and checked data for normality and homogeneity of variance using visual inspection (quantile-quantile plot) and Levene’s test, respectively. We performed a linear mixed effect model (LME; package “nlme” version 3.1.152; Pinheiro et al. 2021) with Bti treatment as a fixed effect and FPM identity as a random effect, followed by an analysis of variance (ANOVA).

**Table 1:** Mean (± 95% confidence interval; CI) NLFA contents (µg per mg dry weight) per fatty acid (FA) and FA group^*a*^ in newt larvae from control (*n*=7) and Bti-treated (*n*=10) FPMs, and results from ANOVA for FA groups

Fatty acids ^a^	Control		Bti		Percent difference in Bti-FPMs compared to controls	*F*_*1,2*_	*p*
	Mean	95% CI	Mean	95% CI			
Σ SFA	3.249	0.730	3.010	0.441	-7.94	0.6910	0.493
16:0	1.851	0.401	1.779	0.301	-4.09		
18:0	1.366	0.365	1.207	0.223	-13.14		
22:0	0.032	0.007	0.024	0.007	-31.97		
Σ MUFA	0.670	0.401	0.704	0.311	4.85	0.025	0.888
16:1n-7	0.258	0.240	0.284	0.175	9.09		
18:1	0.387	0.263	0.401	0.146	3.54		
22:1n-9	0.025	0.006	0.019	0.004	-30.63		
Σ PUFA	1.832	0.605	1.526	0.410	-20.03	1.046	0.414
omega-3 PUFA	0.935	0.344	0.822	0.226	-13.75	0.4559	0.569
18:3n-3 (ALA)	0.407	0.209	0.347	0.142	-17.38		
20:3n-3	0.035	0.019	0.036	0.017	3.18		
22:5n-3 (EPA)	0.436	0.155	0.383	0.098	-13.83		
22:6n-3 (DHA)	0.092	0.048	0.092	0.043	0.14		
omega-6 PUFA	0.767	0.225	0.576	0.162	-33.16	2.134	0.282
18:2n-6 (LIN)	0.258	0.071	0.192	0.087	-34.41		
20:2n-6	0.041	0.032	0.047	0.019	12.54		
20:3n-6	0.044	0.025	0.039	0.016	-14.65		
20:4n-6 (ARA)	0.510	0.170	0.384	0.091	-32.72		
22:2n-6	0.009	0.008	0.006	0.006	-35.52		
Σ Total NLFA	5.751	1.460	5.240	1.022	-9.75	0.4832	0.559

^*a*^Sum (Σ) of saturated fatty acids (SFAs), monounsaturated fatty acids (MUFAs), polyunsaturated fatty acids (PUFAs), omega-3 and omega-6 PUFAs, and total neutral lipid fatty acids (Total NLFAs). Physiologically relevant PUFA eicosapentaeonic acid (EPA), docosahexaenoic acid (DHA), arachidonic acid (ARA), and their precursors linoleic acid (LIN), and alpha-linolenic acid (ALA)

All data visualizations were conducted in R (version 4.1.1) using the packages “ggplot2” (version 3.3.5; Wickham et al. 2016) and “ggpubr” (version 0.4.0; Kassambara 2023).

## Results

### Stable isotopes

The body mass of consumers was similar among control and Bti-treated FPMs (Fig. S1), minimizing potential effects of size-related prey selection (GLMM for newts: degrees of freedom (df) = 7.99, *p* = 0.190; Aeshnidae: df = 8.94, *p* = 0.736).

**Fig. 1:**
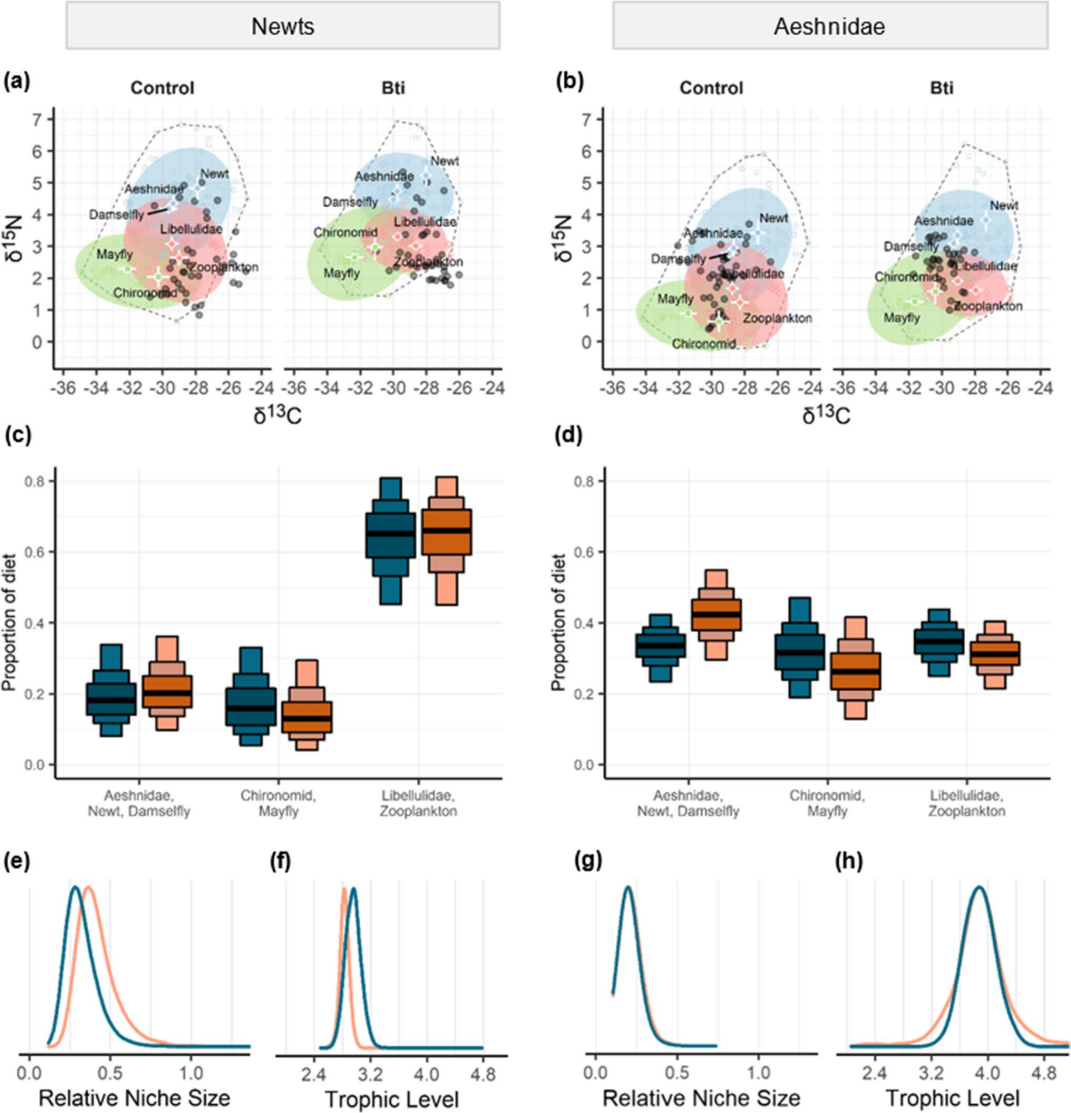
Stable isotope ratios of C and N of newt (**a**) and Aeshnidae larvae (**b**) as consumers shown as black dots, means ± SD, and 95% confidence ellipses of prey organisms (adjusted for trophic enrichment) for each cluster: chironomid and mayfly larvae (green squares and ellipse), Aeshnidae, newt, and damselfly larvae (blue circles and ellipse), Libellulidae larvae, and zooplankton (red diamonds and ellipse). Dotted polygon delineates outer borders of resource polygon. Proportion of diet of newt larvae (**c**) and Aeshnidae larvae (**d**) for each prey cluster, where black lines indicate the medians, and darkest to lightest color of boxes shows Bayesian 50%, 75%, and 95% equal tail credible intervals, respectively. Niche sizes of newts (**e**) and Aeshnidae (**g**) determined from consumer SEA relative to FPM-specific resource SEA, and the trophic position of newts (**f**) and Aeshnidae (**h**) in the food web, in control (dark blue) and Bti-treated (orange) FPMs (*n* = 5)

We determined the following resource clusters used for stable isotope mixing models: Aeshnidae/newt/damselfly, chironomid/mayfly, and Libellulidae/zooplankton. Structures of stable isotopes of the resource clusters were comparable between control and Bti-treated FPMs (Fig. 1a, b) with similar areas of resource polygons (Bayesian median (95% equal tail credible interval): SEA_Control_ = 3.80‰^2^ (3.09–4.47‰^2^) and SEA_Bti_ = 3.50‰^2^ (2.96–5.17 ‰^2^)). In chironomid larvae, we observed approximately 1‰ higher δ^15^N in Bti-treated FPMs compared to control FPMs. According to the mixing model estimates, the diet of newts was dominated by Libellulidae larvae and zooplankton (in both treatments approximately 65%; for values, see Table S3; Fig. 1c). The two other resource clusters Aeshnidae/newt/damselfly and chironomid/mayfly contributed smaller but similar proportions to the diet of newt larvae in control and Bti-FPMs (Fig. 1c). In contrast, the diet of Aeshnidae was balanced between the three resource clusters in control FPMs (~33% each; Fig. 1d). In Bti-FPMs, the diet of Aeshnidae consisted mostly of Aeshnidae/newt/damselfly (~42%), and similar parts of chironomid/mayfly and Libellulidae/zooplankton. The relative niche size calculated by the SEA of the consumer relative to the SEA of the sources was both for control and Bti-treated FPMs similar for newts (SEA_Control_ = 0.301 (0.184-0.562), SEA_Bti_ = 0.393 (0.244–0.711); Fig. 1e) and for Aeshnidae (SEA_Control_ = 0.198 (0.136–0.321); SEA_Bti_ = 0.204 (0.136–0.341); Fig. 1g). The same pattern was observed for the trophic level, where there was no difference between control and Bti-treated FPMs for newts (TP_Control_ = 3.0 (2.8–3.2); TP_Bti_ = 2.8 (2.7–2.9; Fig. 1f) and for Aeshnidae (TP_Control_ = 3.9 (3.5–4.4); TP_Bti_ = 3.8 (2.9–5.1; Fig. 1h). Independent of the treatment, Aeshnidae were approximately one trophic level higher than newts.

### Fatty acids

Neither the body mass (*t*_*15.593*_ = −0.846, *p* = 0.4102) nor the FA content (*F*_*1,2*_ = 0.4832, *p* = 0.559) differed significantly between newts from control and Bti-treated FPMs (Fig. S2).

We determined a total of 15 different FAs in newt larvae (Table 1). The two most abundant FAs were the saturated FAs palmitic acid (16:0) and octadecanoic acid (18:0). Newts from control-FPMs showed approximately 10% higher mean content of total NLFA than Bti-treated newts (Table 1). Although not significant, mean PUFA content was approximately 20% lower in newts from Bti-FPMs compared to controls. This was mainly driven by omega-6 PUFA (Table 1).

## Discussion

### Effects on newts

Contrary to our hypothesis, the results of stable isotope mixing models showed that the Bti treatment had no effect on the diet of newt larvae, despite the observed 41% reduction in chironomid abundance in Bti-FPMs (Gerstle et al. 2023). We hypothesized a diet shift of newts from chironomids towards prey not affected by Bti, such as mayflies (Gerstle et al. 2023; Kolbenschlag et al. 2023a) or zooplankton. Since a clear separation of the isotopic signature of chironomids from other sources was not possible, chironomids and mayflies were grouped into one cluster based on the structure of the resource polygon. Hence, there is the possibility that slight diet shifts between the sources of chironomids, and mayflies could not be detected (Fig. 1a, c). Allgeier et al. (2019b) reported that in a mesocosm experiment, chironomid larvae were the preferred food source for palmate newt larvae over zooplankton, freshwater molluscs, and *Asellus aquaticus*. Despite them recording a reduced abundance of chironomids (53–87%) in Bti-mesocosms compared to controls, these authors also did not observe any differences in estimated diet proportions using stable isotope analyses of C and N. These observations are in accordance with our results, showing that the effect of Bti on the abundance of chironomids (Gerstle et al. 2023) was not reflected in the estimated diet proportions. Interestingly, we determined a higher δ^15^N in larval chironomids from Bti-treated FPMs compared to controls. A similar pattern of higher δ^15^N in emerging chironomids from Bti-treated FPMs was observed in the companion study by Kolbenschlag et al. (2023b). As found by Gerstle et al. (2023), the chironomid subfamily Tanypodinae, which includes predatory species, was less affected by Bti treatment than the predominantly detritivorous Chironominae and Orthocladiinae. This would explain the higher δ^15^N in chironomids from Bti-FPMs since heavier ^15^N isotopes enrich with every trophic level. However, recent results by Röder et al. (2024), who investigated phylogenetic affiliations and ecological traits of chironomid species that emerged from the FPMs in the same year of our study, suggest that feeding type only plays a minor role in the sensitivity towards Bti. Therefore, another possible explanation for the δ^15^N enriched chironomid larvae in Bti-FPMs, not related to the feeding strategy, is that due to increased organic matter from dead chironomids, an additional level at the bottom of the food web is introduced (by an increase in δ^15^N in detritus), similar to observations by McKie et al. (2023).

Approximately 15% and 30% of newt larvae in control and Bti-treated FPMs, respectively, were outside of the resource polygon (Fig. 1a) which indicates that newts could have fed on sources high in δ^13^C and low in δ^15^N. This could be, for instance, periphyton-feeders such as oligochaete worms (Tubificina) or molluscs, which we could not include in our modelling due to the low and uneven sample sizes between FPMs. Additionally, we used TEF values from literature (i.e., skin tissue of adult frogs; Cloyed et al. 2015), whose application has been reviewed in Stephens et al. (2023). These authors pointed out that TEFs are strongly diet- and tissue-specific and there is still a lack of TEFs determined specifically for amphibians and insects. To the best of our knowledge, there is no TEF value that was specifically determined for newt larvae or muscle tissue of amphibians. Therefore, it is possible that the actual newt TEFs deviate from the TEFs applied in our study which would change the position of newts in the resource polygon (Fig. 1a). Although these shortcomings can affect the precision of dietary estimates, relative differences between control and Bti-FPMs can theoretically still be detected. Since both the dietary niche sizes calculated by the SEA (consumer SEA relative to source SEA; Fig. 1e) as well as the trophic level (Fig. 1f) did not notably differ, our results suggest no differences in the diet of newts from control and Bti-FPMs according to stable isotope analyses.

Despite no detected differences in newt’s diet using stable isotope analyses, FA analyses revealed lower content of total NLFAs (~10%) in newts from Bti-treated FPMs compared to controls. Although not significant, we observed the highest difference in omega-6 PUFAs with approximately 30% lower content in larvae from Bti-FPMs (Table 1). This finding suggests that there may be slight differences in newts’ diets that have not been detected by stable isotope analyses and are more likely based on differences in the quality of the consumed prey. FA composition strongly depends on the nutritional quality of the consumers’ diet (e.g., Pietz et al. 2023), except for a few exceptions in which consumers have the capacity to modify dietary FA from precursors (Twining et al. 2016, 2021). The observed lower content of total NLFAs and PUFAs in Bti-treated newts may suggest that when exposed to Bti, newts either consumed prey with lower PUFA content and/or may have experienced a higher energy cost and thus increased usage of storage lipids (i.e., NFLAs). In Bti-treated FPMs, the reduced chironomid availability might have also increased the predatory avoidance (Pérez-Tris et al. 2004), e.g., escaping from Aeshnidae, for which the reduction of chironomids likely caused an increased intake of newts in their diet (see section 4.2).

### Effects on *Aeshnidae*

Aeshnidae consumed a higher proportion of large prey items, like Aeshnidae, newts, or damselflies in Bti-FPMs compared to control-FPMs. As hypothesized, the decreased chironomid abundance has led to increased intraguild predation and/or cannibalism (Gerstle et al. 2023). The more even proportions of different resources in the diet of Aeshnidae (Fig. 1d) suggest a more diverse feeding behavior than newts; however, the analysis of the relative niche size (Fig. 1g) points to a more specialized feeding of Aeshnidae and no effect of Bti on the niche size. Compared to newts, Aeshnidae are approximately one trophic level higher (Fig. 1h), which could also be influenced by the application of different consumer-specific TEFs, as discussed above. Still, Aeshnidae are considered top predators in fishless ponds, while newts and salamander larvae are intermediate predators, likely also due to the smaller body size compared to Aeshnidae (Stemp et al. 2021; Wilbur 1997). Depending on the larval stage and body size of consumers, Aeshnidae function both as predator and prey (Wilbur 1997) and are also known to be cannibalistic under limited prey availability (Vaissi & Sharifi 2016; Van Buskirk 1989), possibly leading to increased intraguild predation and a higher trophic level in the food web compared to other predators like newts. Results from Allgeier et al. (2019b) recorded 27% lower newt survival in mesocosms exposed to Bti, suggesting intraguild predation of newts by Aeshnidae. Also, in a companion study, Gerstle et al. (2023) observed a reduced emergence of Libellulidae and damselflies from Bti-treated FPMs, but no difference in Aeshnidae. This result would be compatible with a diet shift of Aeshnidae in Bti-treated FPMs away from chironomids and towards other prey, for instance, newt larvae.

## Conclusion

The results of this study suggest potential bottom-up shifts in aquatic food webs induced by Bti, as shown for Aeshnidae but not for newt larvae. However, our observations demonstrate the challenges of solely using bulk stable isotope analyses to study aquatic systems and consequently suggest the complementary use with compound-specific stable isotope analysis of fatty acids (Twining et al. 2020) and gut content metabarcoding investigations (Sheppard et al. 2005). This would allow for a more sensitive assessment of predators’ diets, trophic interactions, and energy flow. In our study, the extension by fatty acid analyses for newt larvae pointed to differences in their diet and/or energy budget. Despite being not statistically significant, the observed effect sizes may potentially be relevant for energy and nutrient transfer along the food web within and across aquatic-terrestrial ecosystem boundaries.

### Supplementary Information

Below is the link to the electronic supplementary material.

Supplementary file1 (DOCX 428 KB)

## Data Availability

Contact the corresponding author for data requests.
