# Supplementary material for: Trophic effects of Bti-based mosquito control on two top predators in floodplain pond mesocosms
Source: Environ Sci Pollut Res Int. 2024 Jul 5;31(33):45485–94. doi: 10.1007/s11356-024-34124-w (PMC11269390; doi:10.1007/s11356-024-34124-w)
Supplement: Supplementary file 1 — Supplementary file1 (DOCX 428 KB) [file 11356_2024_34124_MOESM1_ESM.docx]

# Supplementary information

## Dates of flooding and Bti application

Table S 1: Dates of flooding, Bti application and sampling. Application dates were adjusted to weather conditions.

| 11 April 2020 | Flooding, 30>40cm |
| --- | --- |
| 12 April 2020 | Flooding, 40>50cm |
| 14 April 2020 | **1^st^ Bti application** |
| 21 April 2020 | Release water, 50>40cm |
| 22 April 2020 | Release water, 40>30cm |
| 02 May 2020 | Flooding, 30>40cm |
| 03 May 2020 | Flooding, 40>50cm |
| 04 May 2020 | **2^nd^ Bti application** |
| 12 May 2020 | Release water, 50>40cm |
| 13 May 2020 | Release water, 40>30cm |
| 23 May 2020 | Flooding, 30>40cm |
| 24 May 2020 | Flooding, 40>50cm |
| 25 May 2020 | **3^rd^ Bti application** |
| 02 June 2020 | Release water, 50>40cm |
| 03 June 2020 | Release water, 40>30cm |
| 16-19 June 2020 | **Sampling** |

## Dry mass of newts and Aeshnidae used for stable isotope analyses


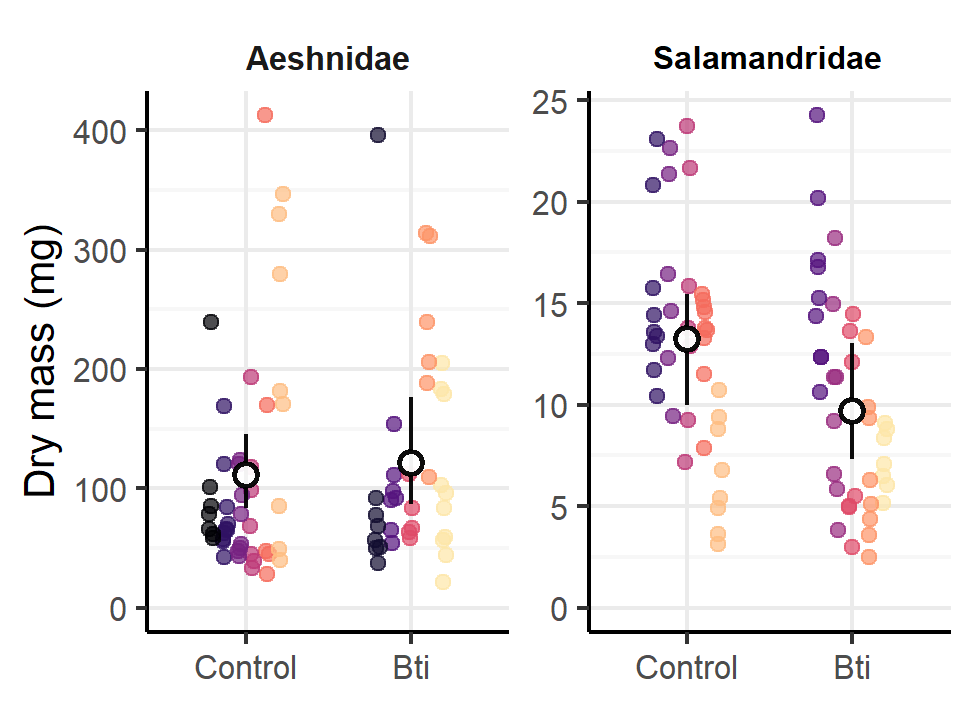


Figure S 1: Dry body mass (mg) of Aeshnidae (left; F_1,8.51_ = 0.122; p = 0.74) and newts (right; F_1,8.18_ = 1.78; p = 0.22). No significant differences between control and Bti-FPMs according to GLMM. Different colors indicate the FPM.

## Sample size used for stable isotope analyses

Table S 2: Sample size (n) per taxa and FPM (even numbers indicate Bti-treated FPMs) for stable isotope analyses, taxa which are not available in some FPMs are denoted with n.a.

| FPM | | | Aeshnidae | | chironomids | | Mayfly | | Libellulidae | newts | | Zooplankton | | Damselfly | |
| --- | --- | --- | --- | --- | --- | --- | --- | --- | --- | --- | --- | --- | --- | --- | --- |
| 1 | | | 7 | | 3 | | 5 | | 10 | n.a. | | 5 | | 5 | |
| 2 | | | 8 | | 5 | | 5 | | 10 | n.a. | | 3 | | 5 | |
| 3 | | | 10 | | 1 | | 5 | | n.a. | 9 | | 5 | | 5 | |
| 4 | | | 7 | | 1 | | 5 | | 10 | 9 | | 5 | | 5 | |
| 5 | | | 8 | | 5 | | 5 | | 10 | 7 | | 5 | | 5 | |
| 6 | | | n.a. | | 5 | | 5 | | n.a. | 8 | | 5 | | 5 | |
| 7 | | | 7 | | 4 | | 5 | | 8 | 7 | | 5 | | 5 | |
| 8 | | | 5 | | 3 | | 5 | | 10 | 7 | | 5 | | 5 | |
| 9 | | | 5 | | 5 | | 5 | | 6 | 10 | | 5 | | 5 | |
| 10 | | | 6 | | 5 | | 5 | | 9 | 9 | | 5 | | 6 | |
| 11 | 8 | 5 | | 5 | | 10 | | 10 | | | 5 | | 6 | |  |
| 12 | 10 | 5 | | 5 | | 6 | | 8 | | | 5 | | 6 | |  |
| n(control) | 45 | 23 | | 30 | | 44 | | 43 | | | 30 | | 31 | |  |
| n(Bti) | 36 | 24 | | 30 | | 45 | | 41 | | | 28 | | 32 | |  |
| Total | 81 | 47 | | 60 | | 89 | | 84 | | | 58 | | 63 | |  |

## Diet proportions of newt and Aeshnidae

Table S 3: Diet proportions of newt and Aeshnidae larvae according to stable isotope mixing models.

| Consumer | Resource cluster | Treatment | Bayesian median | 95% credible interval |
| --- | --- | --- | --- | --- |
| Newt | Aeshnidae/newt/damselfly | Control | 18.9 | 8.0-33.8 |
|  |  | Bti | 20.1 | 9.7-36.2 |
|  | Chironomid/mayfly | Control | 15.7 | 5.3-33.1 |
|  |  | Bti | 12.9 | 4.0-29.4 |
|  | Libellulidae/zooplankton | Control | 65.1 | 45.1-80.9 |
|  |  | Bti | 66.0 | 45.0-81.8 |
| Aeshnidae | Aeshnidae/newt/damselfly | Control | 33.5 | 23.4-42.3 |
|  |  | Bti | 42.4 | 29.6-54.9 |
|  | Chironomid/mayfly | Control | 31.6 | 19.0-47.0 |
|  |  | Bti | 26.2 | 12.9-41.6 |
|  | Libellulidae/zooplankton | Control | 34.7 | 25.0-43.8 |
|  |  | Bti | 31.2 | 21.5-40.4 |

## Dry mass of newts used for fatty acid analyses


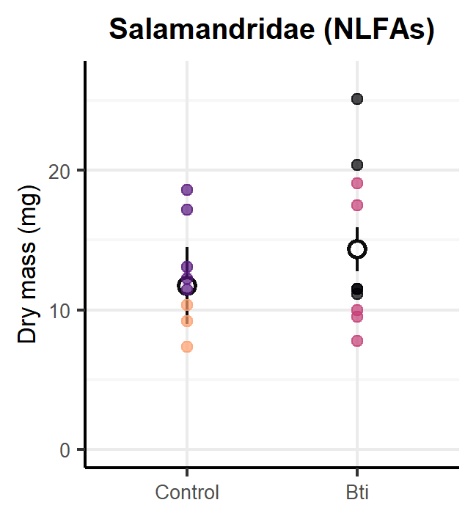


Figure S 2: Dry weight (mg) of newts for NLFA analyses. Different colors indicate different FPMs.
